# Screening effective differential expression genes for hepatic carcinoma with metastasis in the peripheral blood mononuclear cells by RNA-seq

**DOI:** 10.18632/oncotarget.15855

**Published:** 2017-03-02

**Authors:** Yanting Shen, Lu Bu, Rui Li, Zhenzhu Chen, Fei Tian, Na Lu, Qinyu Ge, Yunfei Bai, Zuhong Lu

**Affiliations:** ^1^ Research Center for Learning Science, Southeast University, Nanjing, Jiangsu Province 210096, PR China; ^2^ State Key Laboratory of Bioelectronics, Southeast University, Nanjing, Jiangsu Province 210096, PR China; ^3^ Department of Interventional Radiology, Zhongda Hospital, Medical School of Southeast University, Nanjing, Jiangsu Province 210009, PR China

**Keywords:** hepatic carcinoma, metastasis, PBMC, tumor heterogeneity, diagnosis

## Abstract

Tumor metastasis is a multistep process involving a number of genetic alterations so that the genetic diagnosis is got increasingly attentions today. The aim of this study was to use RNA-seq to screen the effective differential expression genes in the peripheral blood mononuclear cells for the hepatic carcinoma with metastasis. The results showed that hepatic carcinoma samples gathered according to different metastasis. *CCL3*, *CCL3L1*, *JUN*, *IL8*, and *IL1B* were identified in inflammation mediated by chemokine and cytokine signaling pathway (P00031) in the hepatic carcinoma samples with metastasis, and subsequently confirmed by quantitative real-time polymerase chain reaction. In conclusions, *CCL3*, *CCL3L1*, *JUN*, *IL8*, and *IL1B* have the potential to be considered as candidates for future molecular diagnosis of the hepatic carcinoma with metastasis. This work may provide us with new visions into the metastasis process and potential efficient clinical diagnosis in the future.

## INTRODUCTION

Hepatic carcinoma is the fifth most common cancer and the third leading cause of cancer-related death worldwide [1]. Although there are many recent advances in cancer diagnosis and treatment with respect to surgery, radiotherapy, chemotherapy and biotherapy, majority of it remains incurable once it has become metastatic and has a very poor prognosis, primarily due to diagnostic delays or omissions [2–4]. Imaging diagnosis, such as positron emission tomography (PET), is a highly specific tool in liver cancer diagnosis, but in small metastasis or micro-metastasis, typical imaging characteristics are lacking. Some serum markers, such as alpha-fetoprotein (AFP) and alkaline phosphatase (ALP or AKP), are widely used in clinical practice, yet they lacked adequate sensitivity and specificity for the hepatic carcinoma with metastasis. Therefore, seeking effective biomarkers is essential for its diagnosis and treatment.

Metastasis is a complex and multistep process and consists of several stages such as disruption of intercellular adhesion and dispersal of single cells from solid tumor, invasion of blood and lymphatic vessels, immunologic escape in circulation, attachment to endothelial cells, extravasation from blood and lymph vessels, and proliferation and induction of angiogenesis [5–6]. Those processes are interactive with several pathways, suggesting that there is a systematic gene network underlying this process [7]. Thus, the gene expression analysis focusing on this point is got increasingly attentions.

Recently, many related studies have been reported. Zhang et al. [8] identified the dys-regulated genes which were correlated with the venous metastases of hepatocellular carcinoma through large-scale transcriptome analysis by RNA sequencing (RNA-seq). Zhou et al. [9] argued that adrenomedullin played an important role in intrahepatic cholangiocellular carcinoma metastasis, and that adrenomedullin signaling of epithelial-mesenchymal transition might represent a valuable therapeutic target in cancer patients. However, these previous studies were not consistent, because most of them used the tumor tissues as materials. It is known that tumor tissue is a complex cellular society containing variety cells, including the cancer cells, fibroblasts, immune cells, endothelial cells, and inflammatory cells etc., which was called “tumor environment” [10–11]. Thus, these variations within a single tumor, referred to as intra-tumor heterogeneity, could be the main reason that caused the previous studies inconsistent and inadequate to draw a robust conclusion. Besides, the variations between patients, referred to as inter-tumor heterogeneity and classically recognized through different morphology types, expression subtypes, histological classification and grade, or paths that tumor cells take on their way to metastases, also confused the findings [12–13]. Although previous researchers made the biomarkers strict for the tumor types, such as histologic type, cell type, or clinical type to reduce the effect caused by tumor heterogeneity, there was still controversy, because the variation did not exist alone in a tumor sample.

To shed light on these inconclusive problems, instead of the tumor tissue, the peripheral blood mononuclear cells (PBMCs), an easily accessible and minimally invasive sample, were used to investigate the gene expression profile among patients with hepatic carcinoma in this study. With simple components, PBMCs would be beneficial for increasing the reliability of the results through decreasing the intra-tumor heterogeneity. Initial screening of dys-regulated mRNAs was conducted using RNA-seq. Then, the correlation analysis (CA) and principal component analysis (PCA) were performed to group the samples according to their similarity on the gene expression, which could decrease the inter-tumor heterogeneity through reducing the dimension of effectors. Finally, the dys-regulated pathways or biological processes were selected and novel potential mRNAs were confirmed by SYBR Green quantitative real-time polymerase chain reaction (qRT-PCR). This work may help to understand the progression of tumor metastasis and provide us with new visions into the metastasis process and potential efficient clinical diagnosis in the future.

## RESULTS

### Baseline characteristics and RNA-seq information of samples

We used paired-end RNA-Seq to present the gene expression profiles of 23 PBMC samples of patients with hepatic carcinoma and 23 PBMC samples of age-matched healthy people (as the control). RNA-Seq generated from 4,513,370 to 69,018,006 raw reads that were aligned to the human reference hg19, representing 1,117,549 to 21,463,998 mapped reads (Supplementary Table 1). The baseline characteristics of the 23 patients with hepatic carcinoma were shown in Table 1, including age, sex, histological classification and grade, TNM staging and anatomic stage, and metastasis.

**Table 1:** Clinicopathological information

Sample number	Sex	Age	TNM staging and anatomic stage		Histological classification	Histologic Grade	Metastases
**G1**	Female	60	T4N0M0	IVa^a^	Intrahepatic Cholangiocellular carcinoma	-	Direct invasion of adjacent organs
**G2**	Female	63	T2N0M1	IVb	-	-	Distant metastases
**G3**	Male	62	T2N0M0	II	-	-	Intrahepatic metastases
**G4**	Female	51	T4N0M0	-^c^	-	-	Direct invasion of adjacent organs
**G5**	Male	37	T2N0M0	II^b^	Hepatocellular carcinoma	G 3~4	Intrahepatic metastases
**G6**	Male	64	T1N0M0	I^d^	-	-	Solitary tumor
**G7**	Male	51	T1N0M0	I^d^	-	-	Solitary tumor
**G8**	Female	59	T1N0M0	I^d^	-	-	Solitary tumor
**G9**	Female	48	T1N0M0	I^a^	Intrahepatic Cholangiocellular carcinoma	-	Solitary tumor
**G10**	Male	47	T2N0M1	IVb^b^	Hepatocellular carcinoma	-	Distant metastases
**G11**	Male	72	T1N0M0	I^a^	Intrahepatic Cholangiocellular carcinoma	-	Solitary tumor
**G12**	Male	64	T1N0M0	I^b^	Hepatocellular carcinoma	G 1	Solitary tumor
**G13**	Female	54	T1N0M0	I^d^	-	-	Solitary tumor
**G14**	Male	50	T2N1M1	IVb^b^	Hepatocellular carcinoma	G 2	Distant metastases
**G15**	Male	91	T1N0M0	I^d^	-	-	Solitary tumor
**G16**	Male	44	T2N0M0	II^b^	Hepatocellular carcinoma	G 3	Intrahepatic metastases
**G17**	Male	47	T1N0M0	I^b^	Hepatocellular carcinoma	G 2	Solitary tumor
**G20**	Male	36	T1N0M1	IVb	-	-	Distant metastases
**G21**	Male	53	T2N0M0	II^d^	-	-	Intrahepatic metastases
**G22**	Male	58	T1N0M0	I^d^	-	-	Solitary tumor
**G23**	Male	67	T2N0M0	II^d^	-	-	Intrahepatic metastases
**G24**	Male	60	T4N1M1	IVb^a^	Intrahepatic Cholangiocellular carcinoma	-	Distant metastases
**G27**	Male	73	T2N0M0	II^b^	Hepatocellular carcinoma	-	Intrahepatic metastases

^a^according to TNM staging and Anatomic stage for Intrahepatic Bile Duct Tumors (7^th^ ed., 2010) supplied by American Joint Committee on Cancer (AJCC);

^b^according to TNM staging and Anatomic stage for Liver Tumors (7^th^ ed., 2010) supplied by American Joint Committee on Cancer (AJCC);

^c^the anatomic stage cannot be assessed;

^d^stage I, II, and IVb of Anatomic stage for Intrahepatic Bile Duct Tumors and Liver Tumors are the same.

“-” means data is not supplied.

### Characterizing the gene expression profiles of the hepatic carcinoma

To gain insights into the characteristics of gene expression profiles of the hepatic carcinoma, PCA and CA were jointly carried out to generate an evaluation of the similarities or dissimilarities of the RNA-seq outputs.

**Figure 1:**
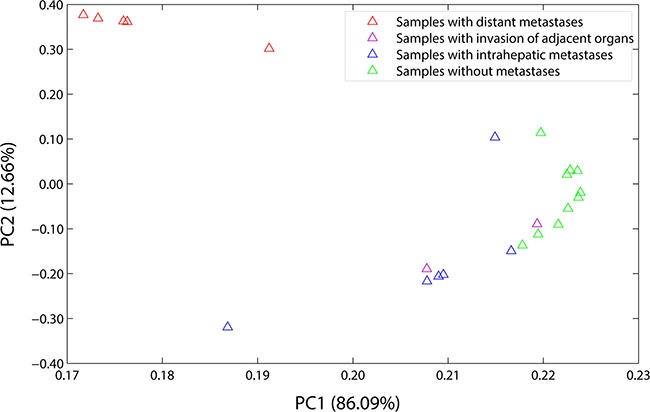
Load plot of PCA PC1 accounted for 86.09% of the overall variance of the data, and PC2 accounted for 12.66%. At PC1, all of the samples were positively related to it, and each of them had the parallel contribution. At PC2, the samples with distant metastases (the red triangles), the samples with intrahepatic metastases (the blue triangles), and the samples without metastases (the green triangles) were gathered into a group, respectively. The samples with distant metastases included G2, G10, G14, G20, and G24. The samples with intrahepatic metastases included G3, G5, G16, G21, G23, and G27. The samples without metastases included G6, G7, G8, G9, G11, G12, G13, G15, G17, and G22. And the samples with invasion of adjacent organs included G1 and G4 (the purple triangles).

PCA is a linear projection method that allows visualization of high-dimensional data in a lower dimensional space. The results of it showed that the first principal component (PC1) accounted for 86.09% of the overall variance of the data, and the second principal component (PC2) accounted for 12.66%. As shown in Figure 1, at PC1, all of the hepatic carcinoma samples were positively related to it, and each of them had the parallel contribution, clearly demonstrating that they were very similar to each other, and the PC1 reflected general characters of gene expression profiles of the hepatic carcinoma; at PC2, the hepatic carcinoma samples with distant metastases (G2, G10, G14, G20, and G24), the hepatic carcinoma samples with intrahepatic metastases (G3, G5, G16, G21, G23, and G27), and the hepatic carcinoma samples without metastases (G6, G7, G8, G9, G11, G12, G13, G15, G17, and G22) were gathered into a group, respectively, indicating that there were differences in gene expression profiles between them, and the PC2 presented the special characteristics of gene expression profiles of the hepatic carcinoma with metastasis. The characteristic values were shown in Supplementary Table 2, and the load coefficients were shown in Supplementary Table 3.

**Figure 2:**
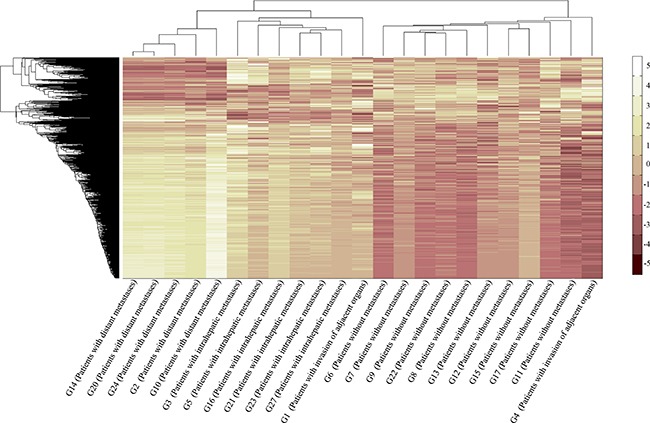
Plot of CA CA was performed using an agglomerative hierarchical clustering algorithm. G2, G10, G14, G20, and G24 were the samples with distant metastases. G3, G5, G16, G21, G23, and G27 were the samples with intrahepatic metastases. G6, G7, G8, G9, G11, G12, G13, G15, G17, and G22 were the samples without metastases. G1 and G4 were the samples with invasion of adjacent organs.

CA is a multivariate statistical technique used to group elements (or variables) and try to achieve maximum homogeneity in each group as well as the biggest difference between them. It was performed using an agglomerative hierarchical clustering algorithm in the present study, and the results were shown in Figure 2. At first, as with the result of PCA, the hepatic carcinoma samples were gathered according to the tumor metastasis rather than the histological classification or grade. And then, the samples with distant metastases and the samples with intrahepatic metastases clustered together. It demonstrated that there was something similar between them in terms of gene expression, which would be used to distinguish the hepatic carcinoma with metastasis from that without metastasis or healthy people. The results of G16, G1, and G4 were abnormal, thus, they were not included in the subsequent analyses.

**Figure 3:**
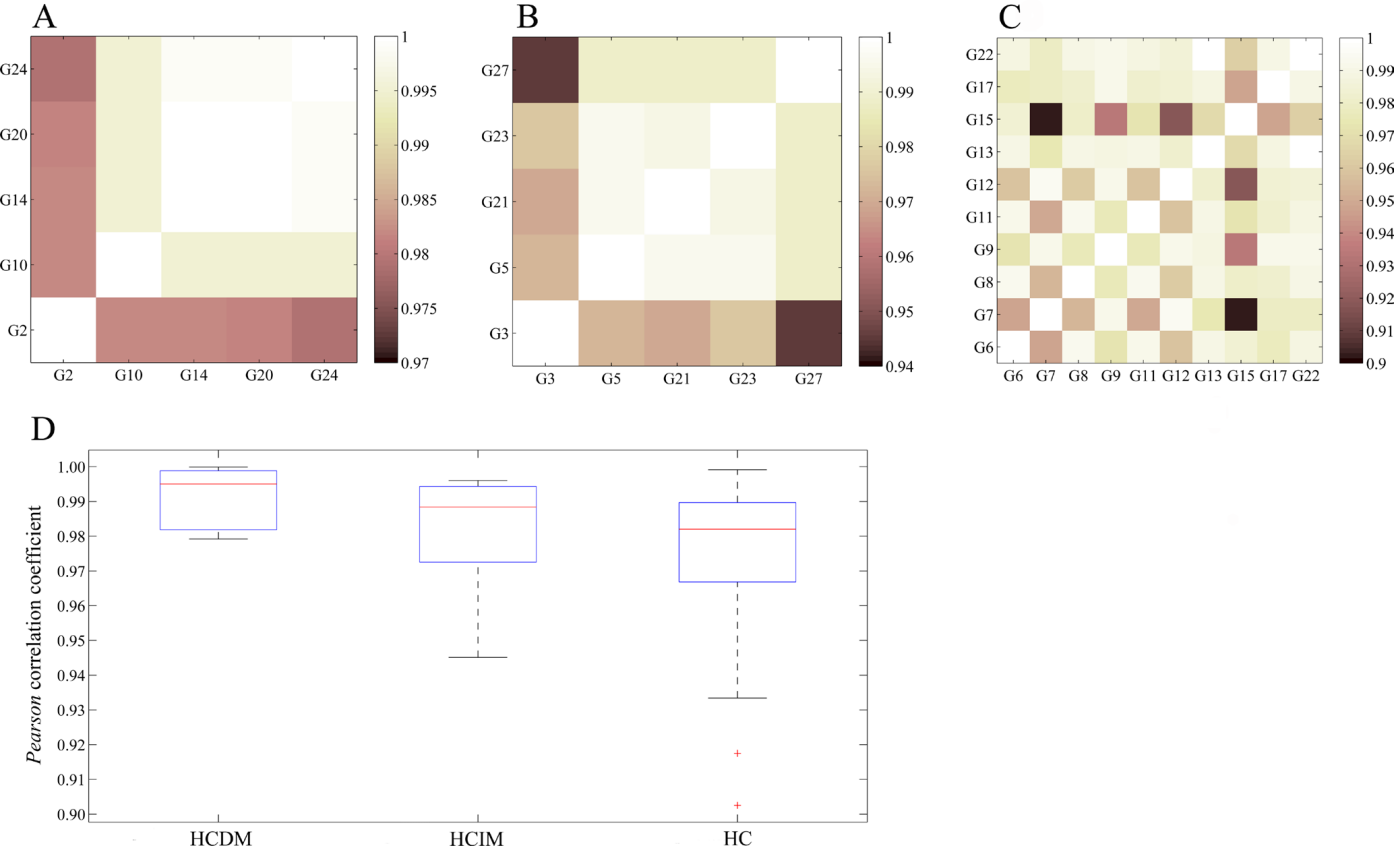
Homogeneity of each phenotype of hepatic carcinoma (**A**) *Pearson* correlation of HCDM; (**B**) *Pearson* correlation of HCIM; (**C**) *Pearson* correlation of HC; (**D**) box plot. One-way ANOVA was performed, and no significant difference was found among HCDM, HCIM, and HC (F = 0.641, *P* = 0.531).

We next divided these hepatic carcinoma samples into three groups: the group of hepatic carcinoma with distant metastases (HCDM), the group of hepatic carcinoma with intrahepatic metastases (HCIM), and the group of hepatic carcinoma without metastases (HC). We computed the whole transcriptome correlations for each group. As anticipated, the gene expression profiles of these three groups were very homogeneous (the average *Pearson* correlation coefficient of each group was 0.9839 ± 0.0063 (HCDM), 0.9821 ± 0.0129 (HCIM), and 0.9753 ± 0.0194 (HC)) (Figure 3A–3C, and Supplementary Tables 4, 5, 6), and there was no significant difference among them (One-way ANOVA: F = 0.641, *P* = 0.531) (Figure 3D), reflecting a well-recognized homogeneity of each phenotype of hepatic carcinoma.

**Table 4:** PANTHER pathways

PANTHER Pathways	Homo sapiens - REFLIST (20814)	Number of DEGs	Expected over/under		Fold enrichment	*P*-value
**HCIM vs. healthy samples**						
Inflammation mediated by chemokine and cytokine signaling pathway (P00031)	245	6	0.15	+	39.21	6.55E-07
CCKR signaling map (P06959)	169	4	0.11	+	37.9	4.51E-04
**HCDM vs. healthy samples**						
Inflammation mediated by chemokine and cytokine signaling pathway (P00031)	245	9	0.8	+	11.24	1.76E-05

**Table 5:** Sequences of primers

Name of primers	Sequences (5′ to 3′)
*CCL3*	F: AGTTCTCTGCATCACTTGCTG
	R: CGGCTTCGCTTGGTTAGGAA
*CCL3L1*	F: CACCTCCCGACAGATTCCAC
	R: GGTCACTGACGTATTTCTGGAC
*JUN*	F: TCCAAGTGCCGAAAAAGGAAG
	R: CGAGTTCTGAGCTTTCAAGGT
*IL8*	F: TTTTGCCAAGGAGTGCTAAAGA
	R:AACCCTCTGCACCCAGTTTTC
*IL1B*	F: AGCTACGAATCTCCGACCAC
	R:CGTTATCCCATGTGTCGAAGAA
	R: CCAGGACCTCATAGCAAACTG
*ACTB*	F: CATGTACGTTGCTATCCAGGC
	R: CTCCTTAATGTCACGCACGAT

### Analysis of the differential expression genes between healthy samples and HCDM, HCIM, and HC, respectively

**Figure 4:**
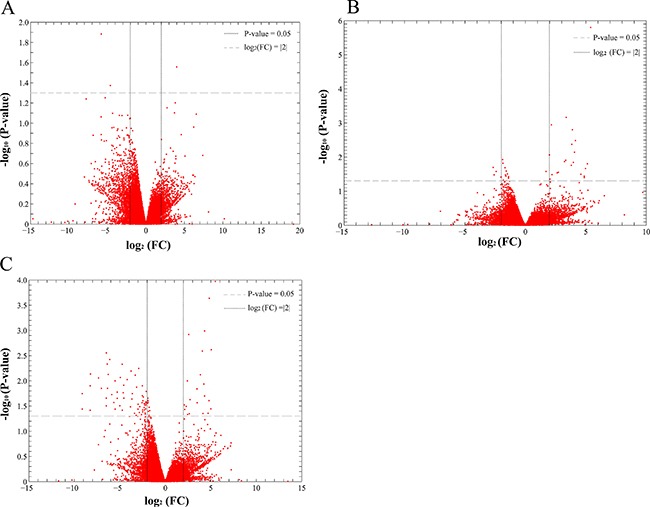
Scatter plot of differential gene expression FC presented the gene expression level of the healthy people vs. the patients. Thus, when the log_2_ (FC) was less than 0, the DEG was an up-DEG. While, when the log_2_ (FC) was more than 0, the DEG was a down-DEG. (**A**) the healthy people vs. HC; (**B**) the healthy people vs. HCIM; (**C**) the healthy people vs. HCDM.

**Figure 5:**
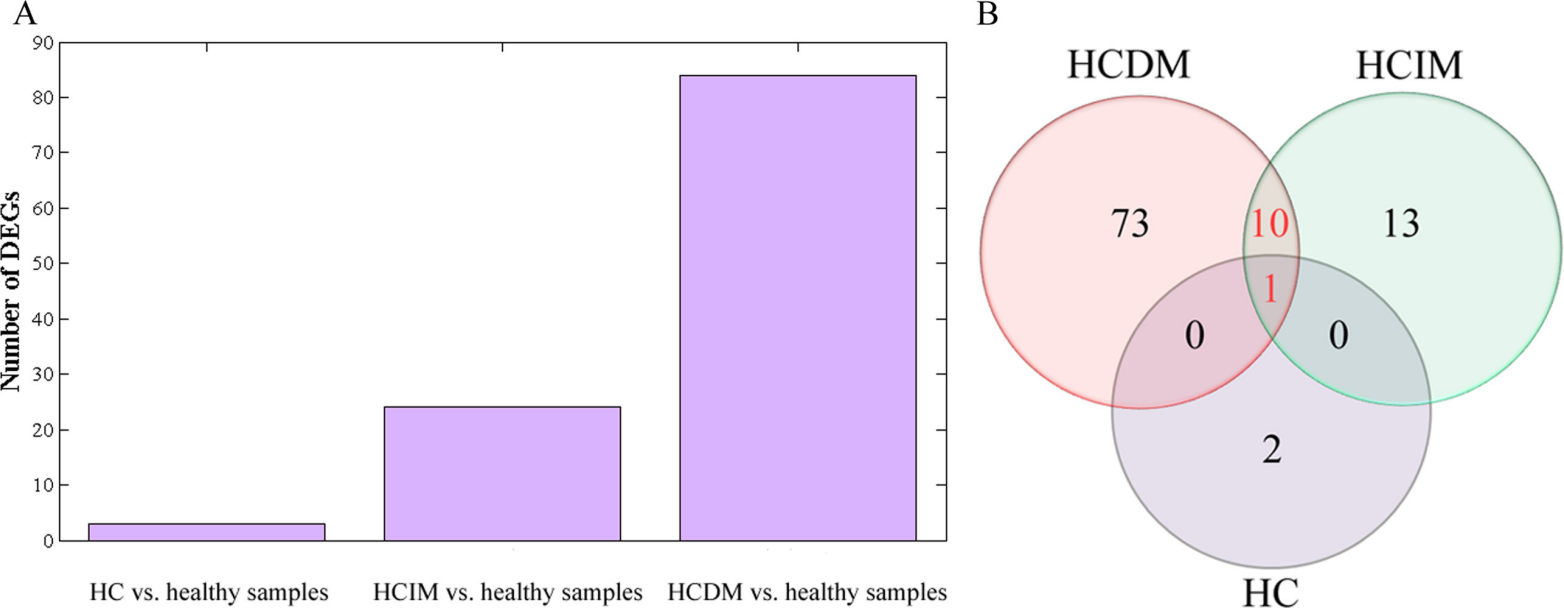
Analysis of differential gene expression (**A**) bar diagram: the number of identified DEGs was specifically increased with the progression of hepatic carcinoma (from HC to HCDM) (*Spearman* correlation coefficient: S = 0.928, *P* < 0.001); (**B**) Venn-Diagram: 1 DEG (*CCL3*) was common to HC, HCIM, and HCDM, which was down-regulated in these three groups, and 10 DEGs were common to HCIM and HCDM, including 6 down-DEGs (*CCL3L1*, *MIR210HG*, *PMAIP1*, *CCL4L1*, *CCL4L2*, *GOS2*,) and 4 up-DEG (*JUN*, *IL1B*, *IL8*, *OSCAR*).

Analysis of DEGs was performed to identify the differential expression genes (DEGs) for HCDM, HCIM, and HC. The results showed that when compared with the healthy samples, 3 DEGs were identified for HC, 24 DEGs were identified for HCIM, and 84 DEGs were identified for HCDM (Figure 4 and Table 2), and the number of identified DEGs was specifically increased with the progression of hepatic carcinoma (from HC to HCDM) (*Spearman* correlation coefficient: S = 0.928, *P value* < 0.001) (Figure 5A). Subsequently, Gene Ontology (GO) analysis was carried out with the PANTHER classification system (http://www.pantherdb.org/) to recognize the functions of the DEGs, and the statistical overrepresentation test was performed to distinguish the significant biological processes and pathways which were involved in the hepatic carcinoma with metastasis. As shown in Tables 3 and 4, we found that HCIM and HCDM shared the same pathologic processes. Two biological processes (immune system process (GO:0002376) and response to stimulus process (GO:0050896)) and one pathway (Inflammation mediated by chemokine and cytokine signaling pathway (P00031)) were found to be common to both of them.

**Table 2:** Different expression genes (log_2_ FC > |2|)

Terms	DEGs	Up-DEGs	Down-DEGs
**HC vs. healthy samples**	3	2	1
**HCIM vs. healthy samples**	24	4	20
**HCDM vs. healthy samples**	84	62	22

**Table 3:** PANTHER GO-Slim biological process

PANTHER GO-Slim Biological Process	Homo sapiens - REFLIST (20814)	Number of DEGs	Expected over/under		Fold enrichment	*P*-value
**HCIM vs. healthy samples**						
behavior (GO:0007610)	20	2	0.01	+	> 100	1.58E-02
immune system process (GO:0002376)	1391	7	0.87	+	8.06	1.57E-03
response to stimulus (GO:0050896)	2170	10	1.36	+	7.38	7.12E-06
**HCDM vs. healthy samples**						
immune response (GO:0006955)	518	12	1.69	+	7.09	2.49E-05
immune system process (GO:0002376)	1391	20	4.54	+	4.4	2.47E-06
response to stimulus (GO:0050896)	2170	20	7.09	+	2.82	2.86E-03

Moreover, we draw a Venn-Diagram to identify the common DGEs. As Figure 5B shown, only 1 DEG (*CCL3*) was common to HC, HCIM, and HCDM, which was down-regulated in these three groups, and 10 DEGs were common to HCIM and HCDM, including 6 down-DEGs (*CCL3L1*, *MIR210HG*, *PMAIP1*, *CCL4L1*, *CCL4L2*, *GOS2*) and 4 up-DEG (*JUN*, *IL1B*, *IL8,OSCAR*). GO analysis was carried out to recognize their functions. Surprisingly, we found that 5 of them, including *CCL3*, *CCL3L1*, *JUN*, *IL8*, *IL1B*, were involved in the inflammation mediated by chemokine and cytokine signaling pathway (P00031), which was shared by HCIM and HCDM, indicating that these 5 DEGs had potential to distinguish the hepatic carcinoma with metastasis from the healthy samples, and among them, *CCL3L1*, *JUN*, *IL1B*, and *IL8*, which were not involved in HC, could be used to distinguish the hepatic carcinoma with metastasis from those without metastasis.

### Validation by qRT-PCR

**Figure 6:**
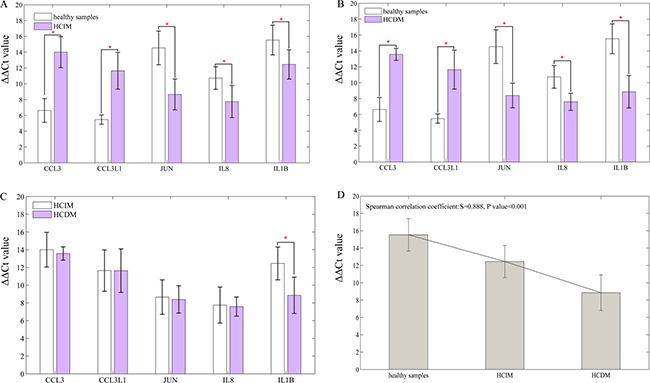
Result of qRT-PCR Data are represented as mean +/− standard deviation. (**A**) healthy samples vs. HCIM: *CCL3* 6.6246 ± 1.51270 vs. 14.0031 ± 1.95683 *P <* 0.001, *CCL3L1* 5.4790 ± 0.59239 vs. 11.6479 ± 2.33065 *P* = 0.003, *JUN* 14.5420 ± 2.12853 vs. 8.6600 ± 1.94387 *P* = 0.002, *IL8* 10.7340 ± 1.43404 vs. 7.7540 ± 2.03102 *P* = 0.028, *IL1B* 15.5340 ± 1.87811 vs. 12.4520 ± 1.85442 *P* = 0.031; (**B**) healthy samples vs. HCDM: *CCL3* 6.6246 ± 1.51270 vs. 13.5701 ± 0.75597 *P <* 0.001, *CCL3L1* 5.4790 ± 0.59239 vs. 11.6377 ± 2.45480 *P* = 0.001, *JUN* 14.5420 ± 2.12853 vs. 8.3920 ± 1.54747 *P* = 0.001, *IL8* 10.7340 ± 1.43404 vs. 7.5860 ± 1.08408 *P* = 0.004, *IL1B* 15.5340 ± 1.87811 vs. 8.8560 ± 2.05059 *P* = 0.001; (**C**) HCIM vs. HCDM: *CCL3* 14.0031 ± 1.95683 vs 13.5701 ± 0.75597 *P* = 0.657, *CCL3L1* 11.6479 ± 2.33065 vs. 11.6377 ± 2.45480 *P* = 0.995, *JUN* 8.6600 ± 1.94387 vs. 8.3920 ± 1.54747 *P* = 0.686, *IL8* 7.7540 ± 2.03102 vs. 7.5860 ± 1.08408 *P* = 0.876, *IL1B* 12.4520 ± 1.85442 vs. 8.8560 ± 2.05059 *P* = 0.020; (**D**) bar diagram: the level of IL1B was specifically decreased with the progression of hepatic carcinoma (from HCIM to HCDM) (*Spearman* correlation coefficient: S = 0.888, *P <* 0.001). ‘*’ presents that there is a significant difference between the groups.

RT-qPCR was performed to further validate these 5 DEGs, and the result was the same with that of RNA-seq (healthy samples vs. HCIM: *CCL3* 6.6246 ± 1.51270 vs. 14.0031 ± 1.95683 *P <* 0.001, *CCL3L1* 5.4790 ± 0.59239 vs. 11.6479 ± 2.33065 *P* = 0.003, *JUN* 14.5420 ± 2.12853 vs. 8.6600 ± 1.94387 *P* = 0.002, *IL8* 10.7340 ± 1.43404 vs. 7.7540 ± 2.03102 *P* = 0.028, *IL1B* 15.5340 ± 1.87811 vs. 12.4520 ± 1.85442 *P* = 0.031; healthy samples vs. HCDM: *CCL3* 6.6246 ± 1.51270 vs. 13.5701 ± 0.75597 *P <* 0.001, *CCL3L1* 5.4790 ± 0.59239 vs. 11.6377 ± 2.45480 *P* = 0.001, *JUN* 14.5420 ± 2.12853 vs. 8.3920 ± 1.54747 *P* = 0.001, *IL8* 10.7340 ± 1.43404 vs. 7.5860 ± 1.08408 *P* = 0.004, *IL1B* 15.5340 ± 1.87811 vs. 8.8560 ± 2.05059 *P* = 0.001) (Figure 6A–6B). Furthermore, the comparison of their expression levels between HCIM and HCDM were conducted, and showed that the expression level of *IL1B* was higher in HCDM than that in HCIM (*P* = 0.02), and showed a positive correlation to the progression of hepatic carcinoma (*Spearman* correlation coefficient: S = 0.888, *P value* < 0.001). No significant difference of other 4 mRNAs was found (*P >* 0.05) (Figure 6C–6D). Finally, we used these five selected DEGs (*CCL3*, *CCL3L1*, *JUN*, *IL8*, *IL1B*) to classify the samples. The results showed that *CCL3*, *CCL3L1*, and *JUN* could identify the patients with metastasis from the healthy people, while *IL1B* could optimally classify the disease progression status (Supplementary Table 7).

## DISCUSSION

Tumor metastasis is a multistep process involving a number of genetic alterations, so that the genetic diagnosis is got increasingly attentions today. However, the existed evidences were still inconclusive due to the tumor heterogeneity. In this study, the correlation of the gene expression profiles for HCDM, HCIM, and HC were very homogeneous (Figure 3) and no significant difference was found among them (*P* = 0.531). It was different from the previous study which presented a well-recognized phenotypic heterogeneity of advanced liver cancer (r = 0.78) [14]. The increased homogeneity reported here was mainly benefit from two aspects. Firstly, PBMCs were used to replace the complex tumor tissues, showing an excellent advantage in reducing the intra-tumor heterogeneity due to their relatively simple cell components. Secondly, PCA and CA were performed to group the hepatic carcinoma samples through evaluating the similarities or dissimilarities of their gene expression profiles. And based on their results which suggested that the change in the gene expression profiles caused by tumor metastasis was greater than that caused by histological classification or grade (Figure 1 and Figure 2), the hepatic carcinoma samples were grouped. It could be helpful for decreasing the inter-tumor heterogeneity.

Subsequently, the dys-regulated DEGs of each group were selected. When compared with the healthy samples, 3, 24, and 84 DEGs were identified for HC, HCIM, and HCDM (Figure 4 and Table 2), presenting an increasing number of DEGs with the progression of hepatic carcinoma (*Spearman* correlation coefficient: S = 0.928, *P* value < 0.001) (Figure 5A). These identified DEGs were probably derived from the circulating tumor cells (CTCs), which shed into the vasculature from a primary tumor and circulated through the bloodstream [15]. Previous studies demonstrated that CTCs contained a variety of mRNAs that played vital roles for subsequent growth of additional tumors in vital distant organs, triggering a mechanism for the vast majority of cancer-related deaths [16–18], and their levels gradually increased with the increase in tumor staging [19–20]. It indicated that the increase number of DEGs with the progression of hepatic carcinoma might be caused by the growth of CTCs, and the number of DEGs might be able to be used for supervising the progression of hepatic carcinoma.

Then, GO analysis was performed to identify the functions of the selected DEGs. Three processes were found to be shared by both HCIM and HCDM. They were immune system process (GO:0002376), response to stimulus process (GO:0050896) and inflammation mediated by chemokine and cytokine signaling pathway (P00031) (Tables 3, 4). Among them, only inflammation mediated by chemokine and cytokine signaling pathway (P00031) involved the same DEGs shared by both HCIM and HCDM (Figure 5B), while others did not. It indicated that although HCIM and HCDM experienced some same processes, most of them were regulated by different DEGs. Therefore, the DEGs that were involved in the same process and shared by HCIM and HCDM should be more reliable and suitable for the diagnosis of the hepatic carcinoma with metastases. As is known, inflammation mediated by chemokine and cytokine signaling pathway (P00031) illustrates chemokine-induced adhesion and migration of leukocytes resulting in the infiltration to the tissue and transcriptional activation enabling recruitment of more leukocytes (Supplementary Figure 1). Thus, when the specific chemokines or receptors were dys-regulated, the recruitment of leukocytes would be disordered resulting in promoting the process of tumor metastasis. In this study, 5 DEGs were found in this pathway in both HCIM and HCDM. They were *CCL3*, *CCL3L1*, *JUN*, *IL8*, and *IL1B*.

**Figure 7:**
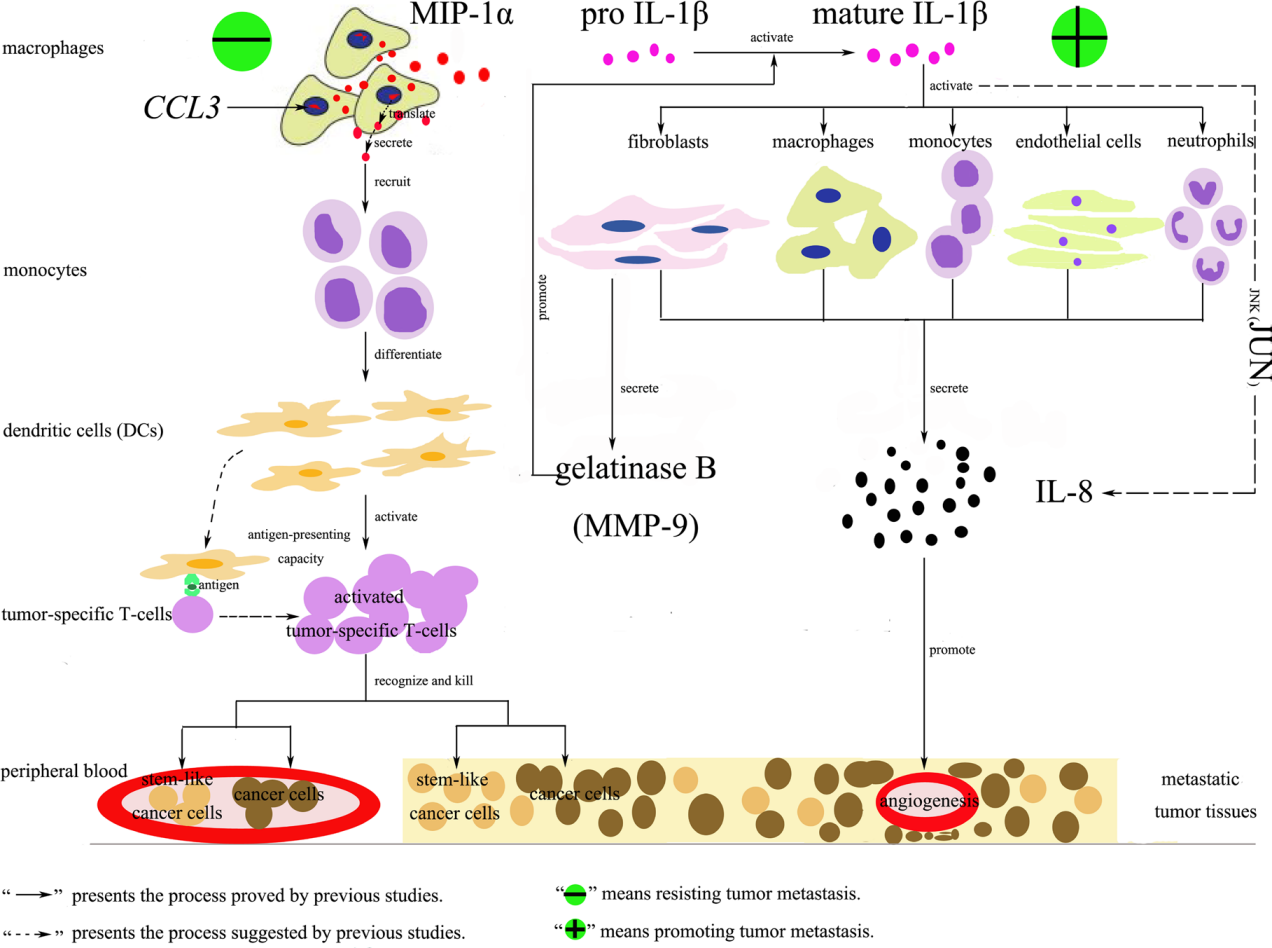
Functional networks of MIP-1α, IL-1β, IL-8 and JUN involved in tumor metastasis There were two processes. The first one could resist tumor metastasis. *CCL3* encoded the macrophage inflammatory proteins-1α (MIP-1α), which was produced by macrophages and had the ability to induce the migration of monocytes. Then the monocytes differentiated into dendritic cells (DCs) to recognize an antigen and activate tumor-specific T-cell responses via their potent antigen-presenting capacity to efficiently recognize and kill stem-like cancer cells [21–26]. The second one could promote tumor metastasis. Mature IL-1β could active macrophages, endothelial cells, monocytes, neutrophils or fibroblasts to secrete IL-8, which had the ability to promote angiogenesis of metastatic tumor tissues. This process might be dependent on the JNK pathway. Besides, the activated fibroblasts by mature IL-1β could secrete gelatinase B (MMP-9), which could promote the conversion of inactive pro IL-1β into mature IL-1β [33].

*CCL3* and *CCL3L1* were found to be down-regulated in the samples of hepatic carcinoma with metastasis. They are located on human chromosome 17 and encode the macrophage inflammatory proteins-1α (MIP-1α) and human CC ligand 3-like protein 1 (CCL3L1), respectively, which belong to the family of chemotactic cytokines known as chemokines. MIP-1α is produced by macrophages and has the ability to induce the migration of monocytes, which then differentiate into dendritic cells (DCs) to recognize an antigen and activate tumor-specific T-cell responses via their potent antigen-presenting capacity to efficiently recognize and kill stem-like cancer cells [21–26]. When the level of MIP-1α decreases, the antitumor activity will be weakened resulting in promoting tumor metastasis. CCL3L1 is the variant of MIP-1α. Previous studies showed that it presented enhanced affinity for C-C chemokine receptor type 5 (CCR5) after cleavage by dipeptidyl peptidase IV (DPPIV)/CD26 and could thereby protect CCR5-expressing cells better against infection with R5 HIV-1 strains [27–28]. However, to our knowledge, no evidence demonstrated its role in tumor metastasis. We hypothesized it might also have the ability of resistant to metastatic oncogenesis, but the further study should be conducted to prove this point. *IL8* was found to be down-regulated in the samples of hepatic carcinoma with metastases in the present study, which was consistent with previous researches. *IL8* is located on human chromosome 4 and encodes interleukin-8 (IL-8), which is produced by macrophages, endothelial cells, monocytes, neutrophils and fibroblasts. It was reported to be correlated with tumor size and tumor stage of hepatocellular carcinoma [29–32]. Thus, when it increased, the angiogenesis would be promoted resulting in hepatic carcinoma cells invasion and metastasis. Besides, in this study, we also found the up-regulated *JUN* and *IL1B* in the samples of hepatic carcinoma with metastases. *JUN* encodes JUN protein, which is the AP-1 transcription factor subunit and involved in the JNK pathway. *IL1B* encodes interleukin-1β protein (IL-1β). It indicated that IL-8 might be mainly induced by IL-1β through the JNK pathway to participate in the process of hepatic carcinoma cells metastasis [33]. The possible functional networks of the proteins encoded by *CCL3*, *CCL3L1*, *JUN*, *IL8*, and *IL1B* were illuminated in Figure 7.

Finally, in order to further validate these 5 DEGs, RT-qPCR were performed and the result was the same with that of RNA-seq, demonstrating that *CCL3*, *CCL3L1*, *JUN*, *IL8*, and *IL1B* were reliable and available to be used as the candidates for hepatic carcinoma with metastasis (Figure 6A and 6B). Furthermore, we found that the expression level of *IL1B* increased with the progression of hepatic carcinoma (Figure 6D), and the results of K-means clustering algorithm showed that *IL1B* could optimally classify the disease progression status, suggesting that it could be used to supervise the progression of hepatic carcinoma.

## MATERIALS AND METHODS

### Patients and sample collection

The fresh EDTA-blood samples were obtained from 28 healthy people and 33 untreated patients with hepatic carcinoma, who visited Zhongda Hospital Affiliated Southeast University (Nanjing, China) and provided written informed consents. Ethics approval was obtained from the Ethics Committee of Zhongda Hospital Affiliated Southeast University. All experiments were performed in accordance with relevant guidelines and regulations set out by the ethical committee.

### Blood processing and RNA extraction

The PBMCs were isolated from 2 ml fresh EDTA-blood of each human subject by Ficoll-Paque™ PREMIUM according to its commercial protocols. And then the PBMC samples were used for RNA extraction using TRIZOL (Invitrogen, Carlsbad, CA) following standard procedures as previously described [34]. RNA quality was accessed by the absorbance at 260 nm (A260) and 280 nm (A280) using NanoDrop ND-1000 (Thermo Fisher Scientific, Waltham, MA), and RNA integrity was determined by RNA integrity number (RIN; Agilent 2100 RIN Beta Version Software).

### RNA-seq

46 RNA samples were selected for sequencing. Among them, 10 were extracted from the hepatic carcinoma patients with solitary tumor, 6 were extracted from the hepatic carcinoma patients with intrahepatic metastases, 5 were extracted from the hepatic carcinoma patients with distant metastases, 2 were extracted from the hepatic carcinoma patients with direct invasion of adjacent organs, and 23 were extracted from the age-matched healthy people. The poly-(A) enriched RNA sequencing libraries were prepared according to a previously published protocol [34], using 0.5 μg of total RNA per library in all instances. Dynabeads mRNA Purification Kit (Ambion, CA) was used to isolate poly-(A) mRNA from total RNA. Random hexamer-primers were used to synthesize first-strand cDNA, while second-strand cDNA was synthesized using buffer, dNTPs, RNase, and DNA polymerase I. Then the double-stranded cDNA fragments were purified using 1.8 x Agencourt AMPure XP Beads(Beckman Coulter, CA), resolved with elution buffer for end reparation and the addition of poly (A), before being ligated to sequencing adapters. Subsequent to fragment selection (about 300bp) using 1.0 x Agencourt AMPure XP Beads (Beckman Coulter, CA), suitable fragments were enriched via PCR amplification, and all of the libraries were multiplexed and sequenced on one lane of an Illumina X10 using standard protocols and reagents.

### Bio-informatic analysis

Raw reads from the image data output from the sequencing machine were generated by Base Calling and saved in FASTQ format. Clean reads were generated by removing reads with adaptors, reads where the number of unknown bases was more than 10%, and low-quality reads (the percentage of the low-quality bases with which value ≤ 5 was more than 50% in one read) using SOAPnuke (version 1.0.1) and then were mapped to the human (hg19) genomes provided by Illumina iGenomes (downloaded from cufflinks.cbcb.umd.edu/igenomes.html) with Tophat2 (version 2.0.7) calling Bowtie2 (version 2.1.0) using the default settings. The alignment and differentially expression genes analysis were performed with Cufflinks (version 2.0.2) [35]. The *q*-value (the false discovery rate (FDR)-adjusted *p-value* [36–38]) ≤ 0.05 and an absolute value of log_2_ fold change (FC) < 2 were used as the thresholds to judge the significance of differences in gene expression.

For functional analyses, GO analysis was carried out with the PANTHER (protein annotation through evolutionary relationship) classification system (http://www.pantherdb.org/) [39]. The statistical overrepresentation test was performed. It was based on the Mann-Whitney test and used to determine whether any ontology class or pathway had numeric values that were non-randomly distributed with respect to the entire list of values.

### qRT-PCR

15 RNA samples were selected for qRT-PCR. Among them, 5 were extracted from the hepatic carcinoma patients with intrahepatic metastases, 5 were extracted from the hepatic carcinoma patients with distant metastases, and 5 were extracted from the age-matched healthy people. For the RT reactions, 1 ul total RNA was used with a PrimeScript™ RT Master Mix (Perfect Real Time) (Takara Bio, Inc.) at 37°C for 15 min and 85 °C for 5 second with a final volume of 10 μl. The following qPCR was performed using SYBR^®^ Premix Ex Taq™ II (Perfect Real Time; Takara, Bio., Inc.) on an Applied Biosystems 7500 real-time PCR machine (Life Technologies) by using 2 μl of the cDNA obtained in the RT reaction. The primers were synthesized by Takara Bio, Inc. and shown in Table 5. The PCR reaction was performed at 95°C for 5 min, followed by 40 cycles at 95°C for 5 sec and 60°C for 31 sec. Each PCR was repeated three times, and the mean value of Ct for each triplicate was calculated. The ΔCt target cDNA was the difference between Ct target cDNA and Ct no template control. And the ΔΔCt value was the difference between ΔCt target cDNA and ΔCt ACTB and used to calculate the amplification fold change in gene expression (ΔΔCt = ΔCt cDNA -ΔCt ACTB; ΔCt cDNA = Ct cDNA – Ct negative reference). The quality of the amplification products was accessed by 2% agarose gel and dissociation curve (Supplementary Figure 2 and Supplementary Figure 3).

### Statistical analysis

All of data were performed with MATLAB^®^ (version 2010b). PCA, CA, *Pearson* correlation analysis was performed to analyze the similarity of the samples, and *Spearman* correlation analysis was performed to analyze the correlations. Venn-Diagrams were generated using the VENNY software (bioinfogp.cnb.csic.es/tools/venny/index.html). Statistical differences were examined by a *t-test* or a one-way ANOVA. K-means clustering algorithm was performed to classify the samples according to the selected DEGs. All statistical tests were performed as two-sided tests. And *P* values < 0.05 were considered statistically significant.

## CONCLUSIONS

To our knowledge, it is first to use the easily accessible and minimally invasive PBMC samples to explore the gene expression profiles of hepatic carcinoma with metastasis. 5 DEGs, including *CCL3*, *CCL3L1*, *JUN*, *IL8*, and *IL1B*, were identified by RNA-seq. They have the potential to be considered as candidates for future molecular diagnosis of the hepatic carcinoma with metastasis. And the number of identified DEGs and the level of *IL1B* presented the potential to be used to supervise the progression of hepatic carcinoma. This work may help to understand the progression of tumor metastasis and provide us with new visions into the metastasis process and potential efficient clinical diagnosis in the future.

## SUPPLEMENTARY MATERIALS FIGURES AND TABLES



## Abbreviations

PET: positron emission tomography
AFP: alpha-fetoprotein
ALP or AKP: alkaline phosphatase
CA: the correlation analysis
PCA: principal component analysis
RNA-seq: RNA sequencing
PBMCs: peripheral blood mononuclear cells
qRT-PCR: SYBR Green quantitative real-time polymerase chain reaction
DEGs: differential expression genes
GO: Gene Ontology
PC: principal component
HCDM: the group of hepatic carcinoma with distant metastases
HCIM: the group of hepatic carcinoma with intrahepatic metastases
HC: the group of hepatic carcinoma without metastases
*CCL3L1*: gene for C-C motif chemokine ligand 3 like 1 protein
*MIR210HG*: MIR210 host gene
*PMAIP1*: gene for phorbol-12-myristate-13-acetate-induced protein
*CCL4L1*: gene for C-C motif chemokine ligand 4 like 1 protein
*CCL4L2*: gene for C-C motif chemokine ligand 4 like 2 protein
*GOS2*: G0/G1 switch 2 gene
*JUN*: JUN proto-oncogene, AP-1 transcription factor subunit
*IL1B*: gene for interleukin 1 beta protein
*IL8*: gene for interleukin 8 protein
*OSCAR*: gene for osteoclast associated, immunoglobulin-like receptor protein
CTCs: circulating tumor cells
MIP-1α: the macrophage inflammatory proteins-1α
CCL3L1: human CC ligand 3-like protein 1
DCs: dendritic cells
CCR5: C-C chemokine receptor type 5
DPPIV: dipeptidyl peptidase IV
IL-8: interleukin-8 protein
JUN: protein encoded by *JUN*
CXCL8: C-X-C motif chemokine ligand 8 protein
IL-1β: interleukin-1β
FDR: the false discovery rate
FC: fold change
