# Supplementary material for: Screening effective differential expression genes for hepatic carcinoma with metastasis in the peripheral blood mononuclear cells by RNA-seq
Source: Oncotarget. 2017 Mar 2;8(17):27976–89. doi: 10.18632/oncotarget.15855 (PMC5438623; doi:10.18632/oncotarget.15855)
Supplement: Supplementary file 1 [file oncotarget-08-27976-s001.pdf]

Screening effective differential expression genes for hepatic carcinoma with metastasis in the peripheral blood mononuclear cells by RNA-seq

Supplementary Materials

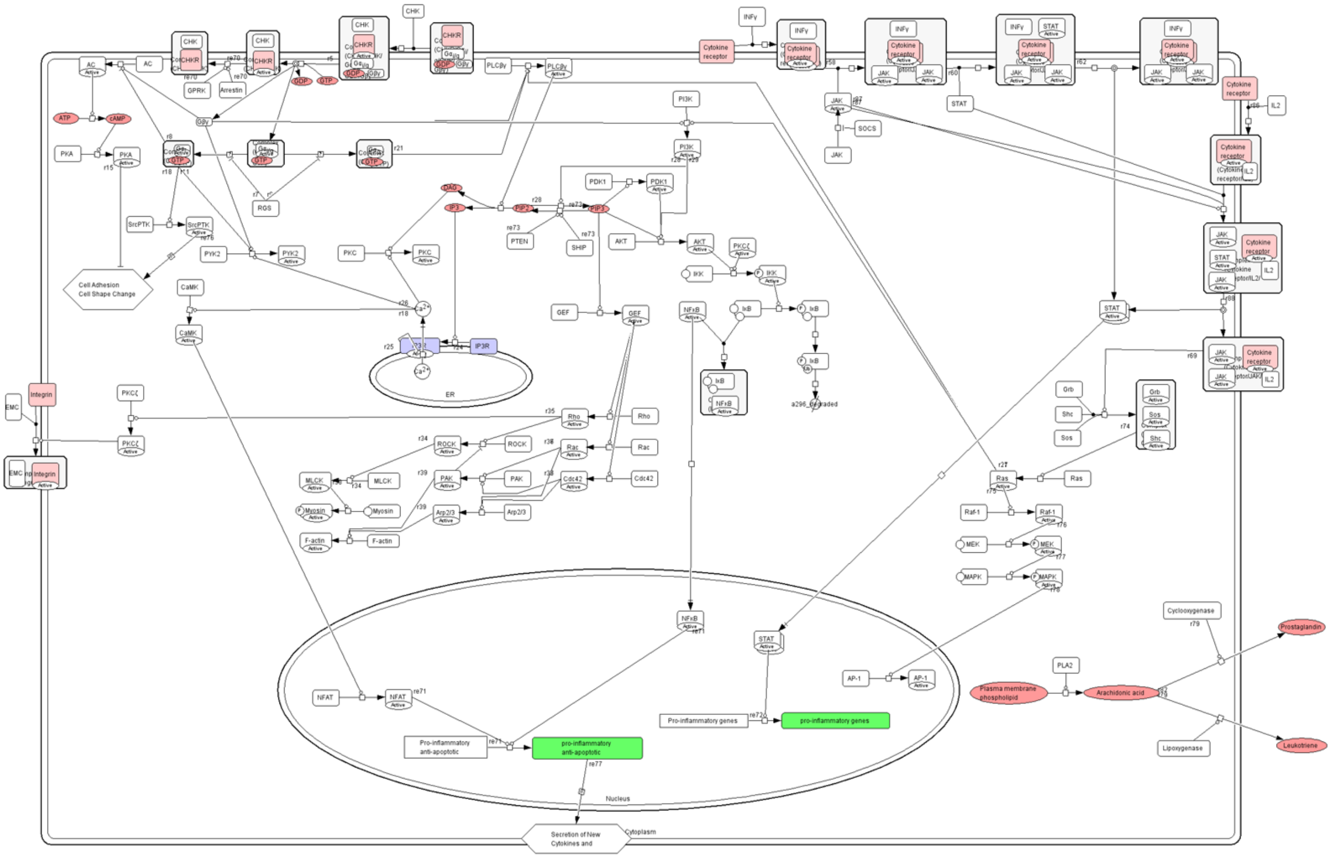

Supplementary Figure 1: Inflammation mediated by chemokine and cytokine signaling pathway (P00031). It was generated by PANTHER (protein annotation through evolutionary relationship) classification system (http://www.pantherdb.org/). This pathway illustrates chemokine-induced adhesion and migration of leukocytes resulting in the infiltration to the tissue and transcriptional activation enabling recruitment of more leukocytes.

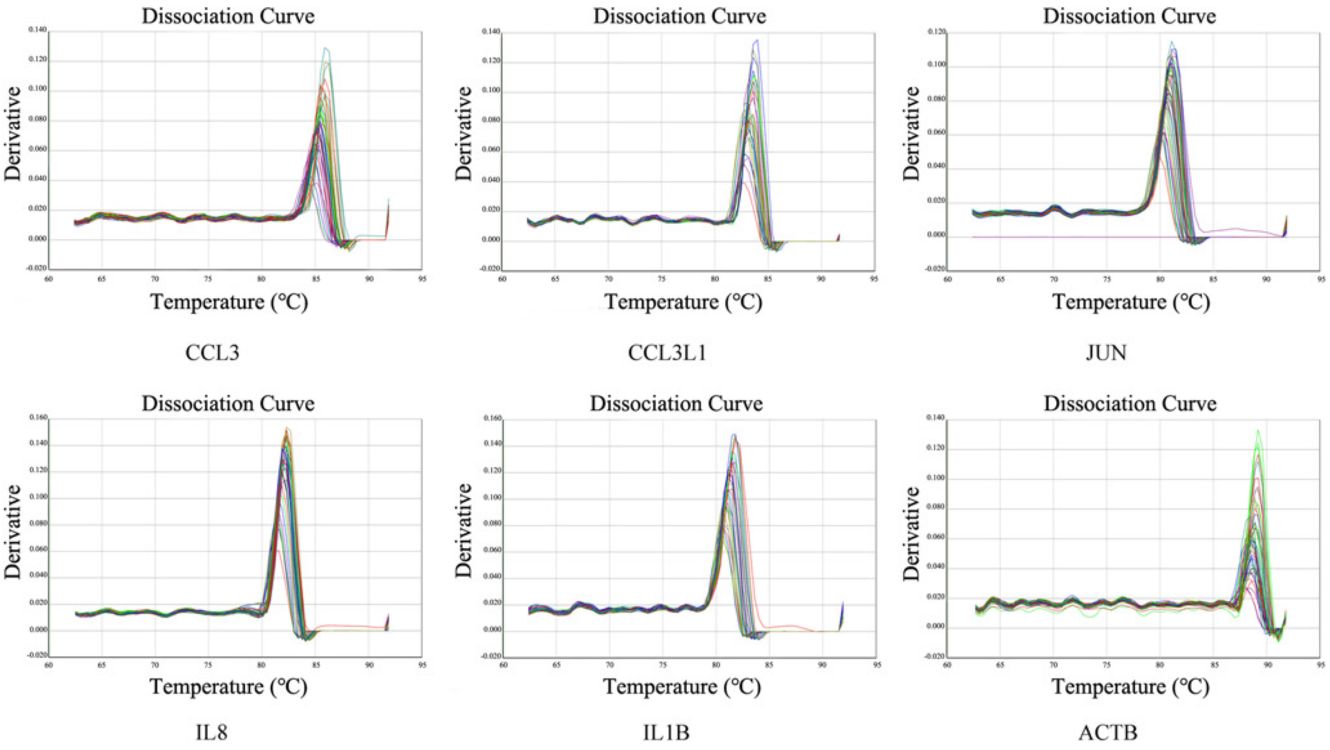

Supplementary Figure 2: Dissociation curves.

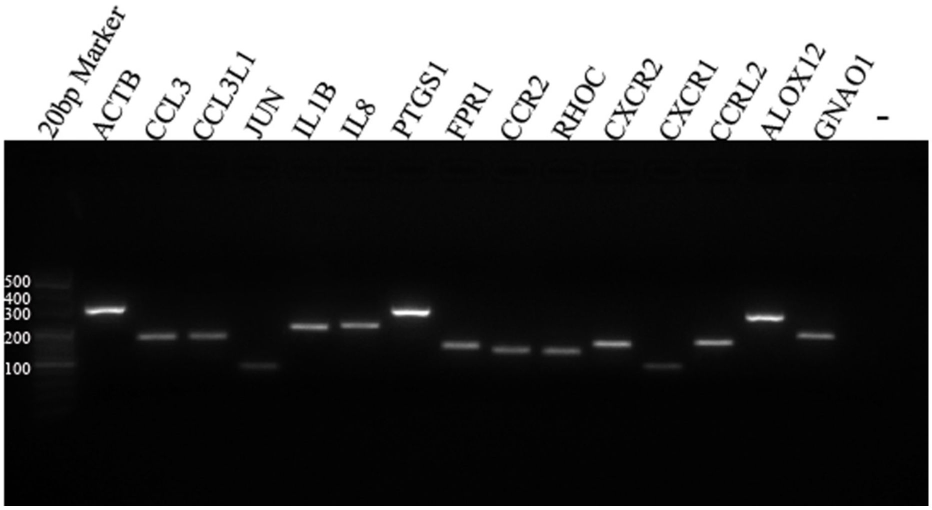

Supplementary Figure 3: Gel electrophoresis.

Supplementary Table 1: Sequencing information

Sample id	Raw reads	Clean reads	Mapped reads
**G1**	52662916	50564762	11283910
**G2**	35491132	32657504	3981833
**G3**	12683916	12155964	3106326
**G4**	34427426	32063042	4694436
**G5**	13427138	12812512	4397933
**G6**	21453450	19801580	4208341
**G7**	22529240	21645250	5968684
**G8**	35366876	33924160	5904985
**G9**	32916410	31430934	7559637
**G10**	45300516	43695342	8634125
**G11**	33557298	32260544	6589066
**G12**	52942250	50777576	10513924
**G13**	33799772	32177562	6484997
**G14**	53024736	49306672	8136421
**G15**	39869740	37917242	7659537
**G16**	36529314	34852836	7139881
**G17**	46524502	43835756	7433911
**G20**	45791264	43947822	8454767
**G21**	44619304	42975018	9192245
**G22**	19799560	18834086	3239819
**G23**	37970048	35637460	6691740
**G24**	45240080	43773214	10619260
**G27**	38975398	37517322	8032727
**B1**	52154374	50339380	15117444
**B2**	69018006	67974762	21463998
**B3**	47850894	47004832	18092885
**B4**	62276294	60108934	17609309
**B5**	46930446	45673178	14119488
**B6**	43877266	41522436	10772146
**B7**	12207972	11674506	4999649
**B8**	16574620	16153582	7835246
**B9**	10139322	9730062	2967694
**B10**	25683026	24993452	9172294
**B11**	4513370	4178354	1117549
**B12**	6487742	6148956	1636077
**B13**	15727034	15215850	3956970
**B14**	24526432	23732784	6721123
**B15**	45995898	44406740	9943348
**B17**	27891544	27085444	6965946
**B18**	59920346	58321410	18384513
**B19**	26521854	25958584	11560105
**B20**	28076680	26078334	4985795
**B21**	42772654	41156312	7024291
**B22**	13625220	12837022	2526358
**B26**	36345060	34883658	6465521
**B31**	21239458	20486552	5526052

Supplementary Table 2: Characteristic values of PCA

Principal Component	Characteristic values	Difference values	Contribution rate	Cumulative contribution rate
**PC1**	19.79991	16.88781	86.08658	86.0865764
**PC2**	2.912107	2.818518	12.66134	98.7479118
**PC3**	0.093589	0.025426	0.406909	99.1548205
**PC4**	0.068163	0.021277	0.296359	99.4511794
**PC5**	0.046885	0.025789	0.203849	99.6550286
**PC6**	0.021096	0.003661	0.091724	99.7467525
**PC7**	0.017436	0.008102	0.075807	99.8225598
**PC8**	0.009334	0.002506	0.040583	99.8631427
**PC9**	0.006828	0.001634	0.029685	99.892828
**PC10**	0.005194	9.27E-05	0.022582	99.9154103
**PC11**	0.005101	0.001952	0.022179	99.9375897
**PC12**	0.003149	0.000346	0.013692	99.9512815
**PC13**	0.002803	0.000699	0.012185	99.9634669
**PC14**	0.002103	0.00075	0.009144	99.9726113
**PC15**	0.001353	0.000145	0.005884	99.9784952
**PC16**	0.001208	0.000117	0.005253	99.9837481
**PC17**	0.001091	0.00031	0.004745	99.9884928
**PC18**	0.000781	0.000116	0.003396	99.9918888
**PC19**	0.000665	0.000123	0.002892	99.9947806
**PC20**	0.000543	0.000185	0.002359	99.9971394
**PC21**	0.000357	0.000107	0.001554	99.9986935
**PC22**	0.00025	0.000199	0.001087	99.9997803

Supplementary Table 3: Load coefficient of PCA

Samples	PC1	PC2
**G3**	0.186861638	–0.319315955
**G2**	0.191225898	0.301827795
**G5**	0.208997807	–0.20596728
**G1**	0.207759523	–0.189530324
**G6**	0.222820395	0.03059253
**G16**	0.214922007	0.103932264
**G24**	0.171694642	0.376874564
**G7**	0.217806487	–0.136802071
**G8**	0.223593288	0.029238935
**G9**	0.221570043	–0.090304356
**G21**	0.209503676	–0.202256619
**G11**	0.222495791	0.020869512
**G12**	0.219457121	–0.112234631
**G10**	0.173246188	0.369192063
**G13**	0.223896426	–0.019241398
**G15**	0.219733629	0.113844198
**G14**	0.176310631	0.361485525
**G20**	0.17591757	0.362662255
**G17**	0.222605165	–0.054885166
**G23**	0.207784867	–0.216944872
**G22**	0.22369821	–0.030317094
**G27**	0.21662541	–0.149486214
**G4**	0.219347017	–0.089355323

Supplementary Table 4: *Pearson* correlation coefficient of HCDM

	G2	G10	G14	G20	G24
**G2**	1	0.981886	0.981865	0.981597	0.979208
**G10**	0.981886	1	0.995004	0.994999	0.995034
**G14**	0.981865	0.995004	1	0.999893	0.998817
**G20**	0.981597	0.994999	0.999893	1	0.998951
**G24**	0.979208	0.995034	0.998817	0.998951	1

Supplementary Table 5: *Pearson* correlation coefficient of HCIM

	G3	G5	G21	G23	G26
**G3**	1	0.972523	0.969727	0.976239	0.9451
**G5**	0.972523	1	0.996039	0.995828	0.988351
**G21**	0.969727	0.996039	1	0.994314	0.988383
**G23**	0.976239	0.995828	0.994314	1	0.988712
**G26**	0.9451	0.988351	0.988383	0.988712	1

Supplementary Table 6: *Pearson* correlation coefficient of HC

	G6	G7	G8	G9	G11	G12	G13	G15	G17	G22
**G6**	1	0.947879	0.993188	0.972419	0.991456	0.959216	0.989377	0.984159	0.977171	0.988512
**G7**	0.947879	1	0.953738	0.991279	0.949806	0.994967	0.974984	0.902546	0.978226	0.978838
**G8**	0.993188	0.953738	1	0.976235	0.993547	0.962226	0.99059	0.980767	0.982672	0.989556
**G9**	0.972419	0.991279	0.976235	1	0.975225	0.991089	0.988183	0.933383	0.991715	0.9907
**G11**	0.991456	0.949806	0.993547	0.975225	1	0.958454	0.989995	0.973183	0.981998	0.989014
**G12**	0.959216	0.994967	0.962226	0.991089	0.958454	1	0.981906	0.917506	0.98331	0.985267
**G13**	0.989377	0.974984	0.99059	0.988183	0.989995	0.981906	1	0.967953	0.988614	0.999122
**G15**	0.984159	0.902546	0.980767	0.933383	0.973183	0.917506	0.967953	1	0.946882	0.96345
**G17**	0.977171	0.978226	0.982672	0.991715	0.981998	0.98331	0.988614	0.946882	1	0.989203
**G22**	0.988512	0.978838	0.989556	0.9907	0.989014	0.985267	0.999122	0.96345	0.989203	1

Supplementary Table 7: Samples classified by selected DEGs

Id	Information of samples	*Group (CCL3)	*Group (CCL3L1)	*Group (JUN)	*Group (IL8)	*Group (IL1B)
**G3**	HCIM	c	b	a	a	b
**G5**	HCIM	c	b	a	a	b
**G16**	HCIM	c	b	a	a	b
**G21**	HCIM	c	b	a	c	b
**G23**	HCIM	c	b	a	c	a
**G2**	HCDM	c	b	a	a	a
**G10**	HCDM	c	b	a	c	a
**G14**	HCDM	c	c	a	c	a
**G20**	HCDM	c	c	a	a	a
**G24**	HCDM	c	c	a	c	a
**B1**	Healthy people	b	a	b	b	c
**B6**	Healthy people	a	a	b	b	c
**B5**	Healthy people	b	a	c	a	c
**B17**	Healthy people	a	a	c	a	c
**B22**	Healthy people	b	a	b	b	c

*"a", "b", "c" represented different groups. If the samples gathered into one group, they would have the same symbol of the group.
